# HERVs may perform as the initial trigger for acquired aplastic anemia

**DOI:** 10.1186/s12967-024-05052-7

**Published:** 2024-03-09

**Authors:** Ting Wang, Nianbin Li, Heng Wu, Rong Fu

**Affiliations:** 1 Department of Hematology, Tianjin Medical University General Hospital, No.154 Anshan Road, Heping District, Tianjin, 300052 China; 2 Tianjin Key Laboratory of Bone Marrow Failure and Malignant Hemopoietic Clone Control, Tianjin Medical University General Hospital, No.154 Anshan Road, Heping District, Tianjin, 300052 China; 3 Tianjin Key Laboratory of Lung Cancer Metastasis and Tumor Microenvironment, Tianjin Lung Cancer Institute, Tianjin Medical University General Hospital, No.154 Anshan Road, Heping District, Tianjin, 300052 China

## To the Editor,

Current evidence indicates that during the onset of acquired aplastic anemia (AA), the characteristic immune state features an increased number and hyperactivity of CD8^+^ T lymphocytes [1]. The reactivation of human endogenous retroviruses (HERVs) promotes the production of viral transcripts and induces the dysregulation of host gene expression and render host cells susceptible to immune attacks [2]. Through single-cell RNA sequencing, we propose that AA may be associated with aberrant expression of HERVs.

**Fig. 1:**
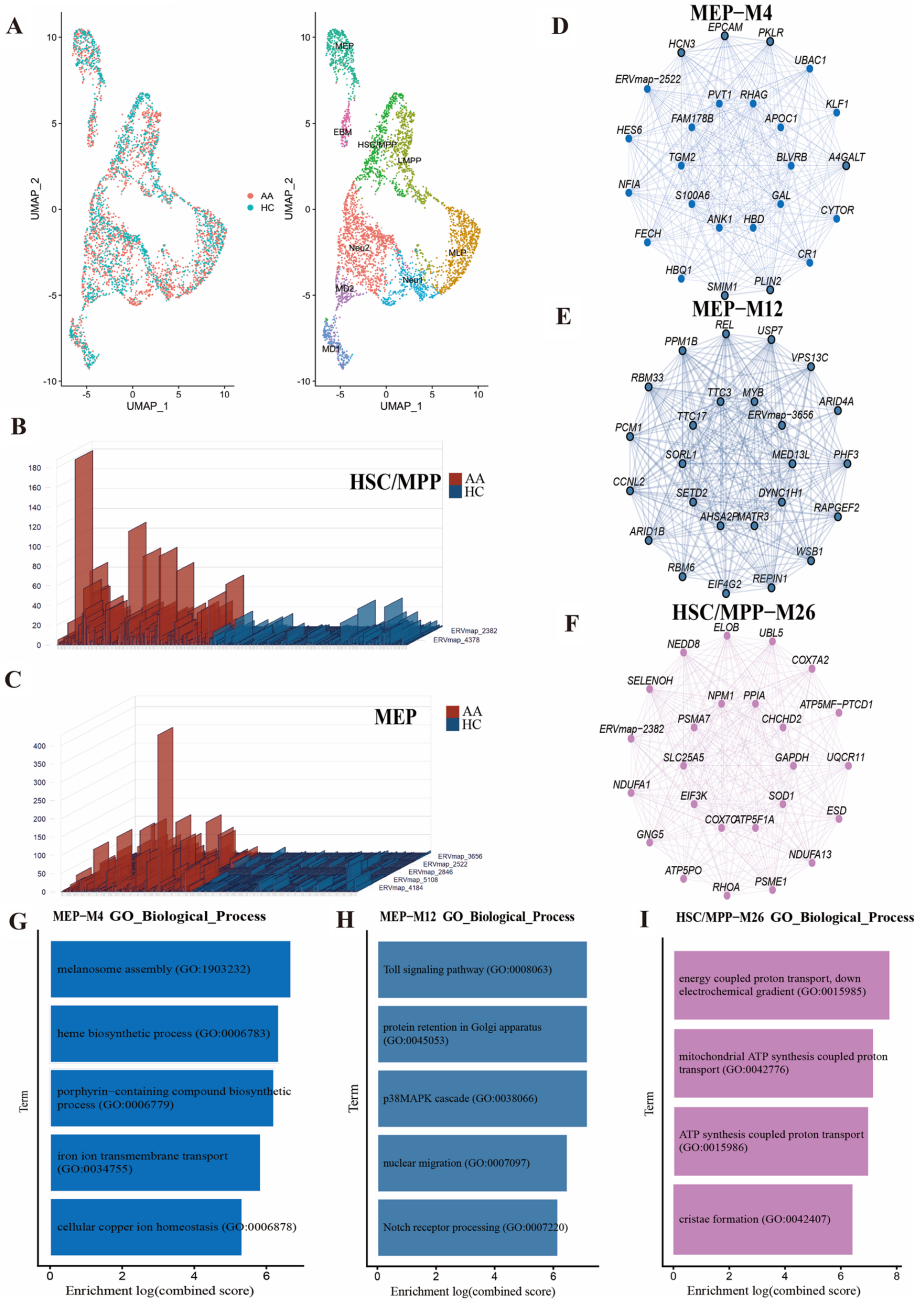
Single-cell landscape and HERVs expression in AA cases and HCs. A UMAP projection of bone marrow HSPCs from AA and HCs, color-coded by cell type. **B, C** Overall expression levels of HERV loci in HSC/MPP and MEP, compared by the Wilcoxon rank-sum test. **D**–**F** Co-expression networks of differentially expressed HERVs in the MEP and HSC/MPP cell clusters. **G**–**I** Biological process enrichment analysis of co-expressed genes of ERVmap_2522, ERVmap_3656, and ERVmap_2382

After preprocessing and quality control, we obtained the expression profiles of 3220 HERVs from ERVmap in hematopoietic stem and progenitor cells (HSPCs) and T cells in AA. 4318 HSPCs were obtained and categorized into 9 distinct HSPCs types (Fig. 1A, Additional file 1: Fig. S1A) [3]. The cell proportions of HSPCs were quantitated in the AA and healthy control (HC) (Additional file 1: Fig. S1B), and the chi-square test was performed to compare the observed and expected cell numbers in various clusters (Additional file 1: Fig. S1C). The results revealed there were notable deficiencies in hematopoietic stem cells and multipotent progenitors (HSCs/MPPs) and megakaryocyte and erythroid progenitors (MEPs) in AA. Significant overexpression of ERVmap_2382 and ERVmap_4378 are detected in the HSC/MPP cluster. ERVmap_3656, ERVmap_2522, ERVmap_2846, ERVmap_5108, and ERVmap_4184, were also overexpressed in the MEP subset (Fig. 1B, C). However, there was no intersection of differentially upregulated HERVs between the HSC/MPP and MEP clusters (Additional file 1: Fig. S1D). Differential analysis of the remaining 7 clusters’ HERVs demonstrated elevated expression of HERVs in the other HSPC clusters of AA(Additional file 1: Fig. S2A–I). To examine the potential impact of HERV expression on genes, a high-dimensional weighted gene co-expression network analysis was performed (Additional file 1: Fig. S3A, B). Highly expressed HERVs in the HSC/MPP and MEP subsets of AA were selected for co-expression analysis. The MEP-M4 and MEP-M12 modules are co-expression modules for ERVmap_2522 and ERVmap_3656, respectively, while the HSC/MPP-M16 module constitutes the co-expression module for ERVmap_2382 (Fig. 1D–F). Biological process enrichment analysis revealed the MEP-M4 module had the highest enrichment score for melanosome assembly, the MEP-M12 module for the Toll and p38MAPK signaling pathway, and the HSC/MPP-M16 module for energy-coupled proton transport down an electrochemical gradient (Fig. 1G–I). These findings suggest aberrant expression of certain HERVs may render HSC/MPP and MEP cell clusters in HSPCs susceptible to immune damage, potentially representing a critical factor in AA pathogenesis [4].

**Fig. 2:**
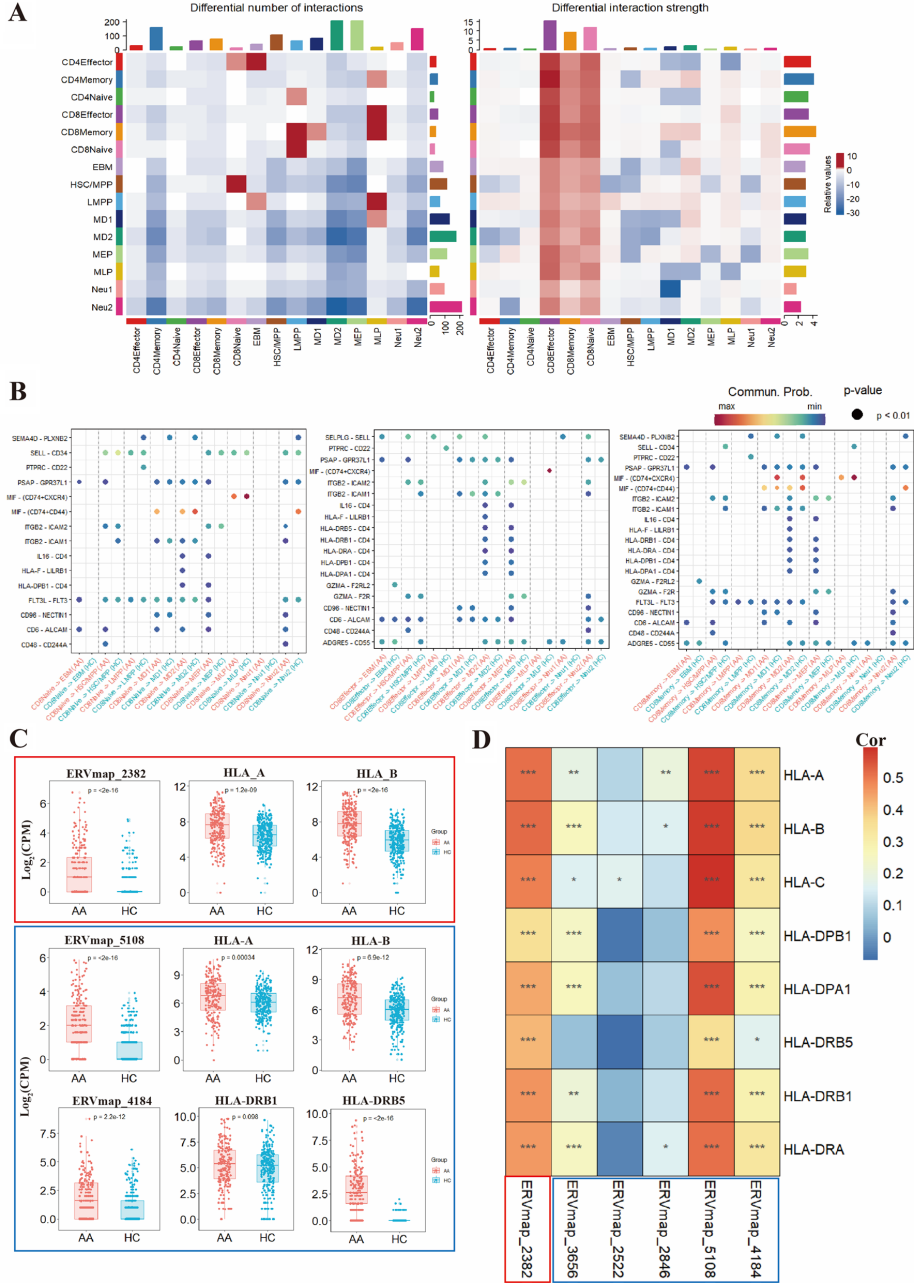
Cell communication between HSPCs and T cells, and expression of HERVs and HLAs. **A** Strengths of signal receptors and transmitters in different pathways of T cells and HSPCs. The colored bar at the top represents the sum of the numerical columns displayed in the heat map (incoming signals). The colored bar on the right represents the sum of a row of values (outgoing signals). In the color bar, red (or blue) indicates an increase (or decrease) of signals in AA compared to HCs. **B** Communication probability between cell clusters. Important ligand-receptor pairs involved in key signaling pathways where CD8^+^ T cells act as signal senders. **C** Box plots showing the consistency of high HERV expression with the expression of HLA loci in the HSC/MPP and MEP cell clusters in AA. The red and blue boxes represent calculations for the HSC/MPP and MEP cell clusters, respectively. **D** Heatmap displaying the correlations between high expression HERVs and HLA loci in the HSC/MPP and MEP cell clusters in AA. Red and blue indicate high and low correlation coefficients, respectively. * *P* < 0.05, ** *P* < 0.01, *** *P* < 0.001. The red and blue boxes for HERVs represent the correlations for the HSC/MPP and MEP cell clusters, respectively

CD4^+^ and CD8^+^ T cells were subdivided into three clusters (Additional file 1: Fig. S4A–F) [3]. Differential expression analysis of HERVs in CD4^+^ T cells and CD8^+^ T cell subsets showed significantly reduced expression of HERVs in the AA group (Additional file 1: Fig. S5A–F). Ligand-receptor interaction analysis revealed a significant increase in the interaction between T cells and HSPCs in AA patients, with notable increasing in both frequency and strength of interactions for CD8^+^ T cells (Fig. 2A). Interactions between CD8^+^ effector T cells and CD48-CD244A, PSAP-GPR37L1, and SELPLG-SELL receptor-ligand pairs in the HSC/MPP cluster were enhanced in AA (*P* < 0.01) (Fig. 2B). CD8^+^ effector T cells in AA exhibited higher interactions with multiple HLA-related receptor-ligand pairs in the MEP cluster, including HLA-DRB1 and CD4, HLA-DPB1 and CD4 (*P* < 0.01) (Fig. 2B). Previous evidence indicates aberrant HLA expression affects immune tolerance in the body and is closely associated with the onset of AA [5]. HLA expression was generally higher in the HSC/MPP and MEP in AA patients, with significantly elevated HLA-DRB5 expression in AA patients (Additional file 1: Fig. S6A). In the MEP cluster, high expression of ERVmap_5108 and ERVmap_4184 in AA patients correlated with HLA-A, HLA-B, HLA-DRB1, and HLA-DRB5 levels (Fig. 2C). The correlations between the highly expressed HERVs and selected HLAs were determined in the HSC/MPP and MEP clusters, revealing a significant positive correlation between the highly expressed HERVs and HLAs (Fig. 2D). These findings suggest HERVs expression may regulate HLAs in HSPCs, modulating the immune attack of T lymphocytes in HSPCs.

To assess whether the impact of HERVs on HSPCs persists throughout their differentiation and development, we demonstrated the distribution of differentially expressed HERVs in the HSC/MPP and MEP clusters along developmental trajectory 3(S7A). ERVmap_2382 expression levels in the developmental stages of HSC/MPP were similar in the AA and HC groups along the cell differentiation trajectory. HERVs, including ERVmap_5108 and ERVmap_4184, exhibited increased expression levels in the MEP developmental stages of AA. In HLA-DRB5, higher expression levels were detected in the HSC/MPP and MEP developmental stages of AA (S7B).

In conclusion,HERVs are upregulated in the HSPCs of AA. First, this affects the transcriptional landscape of HSPCs, affecting energy synthesis and transport, and triggering inflammatory pathways such as the Toll and p38MAPK signaling pathways. Second, activation of HERVs directly or indirectly upregulate HLAs, indirectly mediating T cell activation, which is reflected by significantly enhancing interaction between CD8^+^ T cells and HSPCs in AA. Due to the limited number of cases, HERVs-related factors in AA patients still need to be further studied. The present study boldly proposes HERVs may serve as the initiating antigens triggering AA, providing a novel perspective for unraveling AA pathogenesis.

### Supplementary Information

**Additional file 1:** **Figure S1.** Single-cell landscape and HERVs expression in the AA and HCs. **A **Heatmap illustrating the expression of typical marker genes for major cell types. **B **Relative proportions of major cell types in AA cases and HCs.** C **Dot plot depicting the distribution of major cell types, as estimated by RO/E, across different tissue types. The ratio of observed to expected cell counts is shown for each cell type. **D **Intersection of upregulated and downregulated HERVs in HSC/MPP and MEP cell clusters. **Figure S2.** Volcano plots of differential HERVs in distinct subtypes of HSPCs. **A**–**I **Volcano plots depicting differential expression of HERVs in 9 subtypes of HSPCs. Criteria for selection were *P*<0.05 and log_2_ |fold change| >1. **Figure S3.** Co-expression analysis of HERVs in HSPCs. **A **Determination of the soft-thresholding parameter for co-expression analysis.** B **Construction of a co-expression network based on the optimal soft-threshold, grouping genes and HERVs into distinct modules. **Figure S4.** T cell clustering. **A **UMAP projection of bone marrow CD8^+^ T cells from AA cases and HCs, color-coded by cell type.** B **Heatmap depicting the expression of typical marker genes for major cell types in CD8^+^ T cells.** C **Relative proportions of major cell types in CD8^+^ T cells from AA cases and HCs. **D **UMAP projection of bone marrow CD4^+^ T cells from AA cases and HCs, color-coded by cell type. **E **Heatmap illustrating the expression of typical marker genes for major cell types in CD4^+^ T cells. **F **Relative proportions of major cell types in CD4^+^ T cells from AA cases and HCs. **Figure S5.** Volcano plots of differential HERVs in distinct subtypes of T cells. **A–C **Volcano plots illustrating differential expression of HERVs in 3 subtypes of CD8^+^ T cells. Criteria for selection were *P*<0.05 and log_2_ |fold change| >1.** D**–**F **Volcano plots showing differential expression of HERVs in 3 subtypes of CD4^+^ T cells. Criteria for selection were *P*<0.05 and log_2_ |fold change| >1. **Figure S6.** Correlations between differential HERVs in HSC/MPP and MEP cell clusters and HLA expression in AA. **A **Density plot illustrating the expression densities of HLA-A, HLA-B, HLA-DRB1, and HLA-DRB5 loci in cell clusters from untreated AA patients and control samples. The red and blue boxes indicate the HSC/MPP and MEP cell clusters. **Figure S7.** Pseudotemporal analysis of HERVs and HLA in HSPCs from AA cases and HCs. **A **Pseudotemporal analysis trajectory of HSPCs, with cell subtypes referenced from Fig. 1A.** B **Expression patterns of differentially expressed HERVs in HSC/MPP and MEP from AA cases and HCs along trajectory 3.

## Data Availability

All the data that support the findings of this study are available within the Article and Supplementary Files. Single-cell RNA sequencing raw data have been previously deposited in the Gene Expression Omnibus database under accession number GSE145668. If you have any reasonable questions, please contact the corresponding author (furong8369@tmu.edu.cn).

## References

[1] Ben Hamza A, Welters C, Stadler S, Brüggemann M, Dietze K, Brauns O (2024). Virus-reactive T cells expanded in aplastic anemia eliminate hematopoietic progenitor cells by molecular mimicry. Blood J.

[2] Jakobsson J, Vincendeau M (2022). SnapShot: human endogenous retroviruses. Cell.

[3] Zhu C, Lian Y, Wang C, Wu P, Li X, Gao Y (2021). Single-cell transcriptomics dissects hematopoietic cell destruction and T-cell engagement in aplastic anemia. Blood.

[4] Saini SK, Ørskov AD, Bjerregaard A-M, Unnikrishnan A, Holmberg-Thydén S, Borch A (2020). Human endogenous retroviruses form a reservoir of T cell targets in hematological cancers. Nat Commun.

[5] Pagliuca S, Gurnari C, Hercus C, Hergalant S, Nadarajah N, Wahida A (2022). Molecular landscape of immune pressure and escape in aplastic anemia. Leukemia.

