# Supplementary material for: HERVs may perform as the initial trigger for acquired aplastic anemia
Source: J Transl Med. 2024 Mar 9;22:260. doi: 10.1186/s12967-024-05052-7 (PMC10924994; doi:10.1186/s12967-024-05052-7)
Supplement: Supplementary file 1 — Additional file 1: Figure S1. Single-cell landscape and HERVs expression in the AA and HCs. A Heatmap illustrating the expression of typical marker genes for major cell types. B Relative proportions of major cell types in AA cases and HCs. C Dot plot depicting the distribution of major cell types, as estimated by RO/E, across different tissue types. The ratio of observed to expected cell counts is shown for each cell type. D Intersection of upregulated and downregulated HERVs in HSC/MPP and MEP cell clusters. Figure S2. Volcano plots of differential HERVs in distinct subtypes of HSPCs. A–I Volcano plots depicting differential expression of HERVs in 9 subtypes of HSPCs. Criteria for selection were P<0.05 and log2 |fold change| >1. Figure S3. Co-expression analysis of HERVs in HSPCs. A Determination of the soft-thresholding parameter for co-expression analysis. B Construction of a co-expression network based on the optimal soft-threshold, grouping genes and HERVs into distinct modules. Figure S4. T cell clustering. A UMAP projection of bone marrow CD8+ T cells from AA cases and HCs, color-coded by cell type. B Heatmap depicting the expression of typical marker genes for major cell types in CD8+ T cells. C Relative proportions of major cell types in CD8+ T cells from AA cases and HCs. D UMAP projection of bone marrow CD4+ T cells from AA cases and HCs, color-coded by cell type. E Heatmap illustrating the expression of typical marker genes for major cell types in CD4+ T cells. F Relative proportions of major cell types in CD4+ T cells from AA cases and HCs. Figure S5. Volcano plots of differential HERVs in distinct subtypes of T cells. A–C Volcano plots illustrating differential expression of HERVs in 3 subtypes of CD8+ T cells. Criteria for selection were P<0.05 and log2 |fold change| >1. D–F Volcano plots showing differential expression of HERVs in 3 subtypes of CD4+ T cells. Criteria for selection were P<0.05 and log2 |fold change| >1. Figure S6. Correlations between [file 12967_2024_5052_MOESM1_ESM.docx]

HERV is the initial trigger for acquired aplastic anemia

Methods： Pages 2-4

Reference： Page 5

Figures： Pages 6-14

**Methods S1：**

1. Public dataset acquisition

A total of 16 bone marrow samples were collected from patients with acquired aplastic anemia (AA) (P1-P16) and 3 bone marrow samples from healthy controls (HCs) (Ctrl1, Ctrl2, Ctrl4) in the Gene Expression Omnibus (GEO) dataset GSE145668. 3' end mRNA single-cell RNA sequencing (scRNA-seq) data were obtained and converted from SRA files to FASTQ files with the SRA Toolkit (https://github.com/ncbi/sra-tools). Detailed information about the samples and sequencing methods can be found in the GSE145668 dataset[1]. A total of 3220 Human endogenous retroviruses (HERVs) proviral loci were obtained from the ERVmap database[2]. We included HERVs sites with unique chromosomal positions and did not intentionally exclude any sites. The human genome hg38 (https://hgdownload.soe.ucsc.edu/goldenPath/hg38/bigZips/) was employed for gene comparison. We aligned the 3220 HERV loci from the ERVmap database and human genes from UCSC to the 3' end mRNA single-cell RNA sequencing (scRNA-seq) data of the AA and HC groups. The resulting expression matrices for HERVs and genes in the AA and HC groups were used for subsequent analysis.

2. Pipeline for the detection of HERVs

Initially, FastQC (version 0.11.9) was used to assess the quality of FASTQ files and furnish a swift overview of sequencing quality[3]. Cutadapt (version4.0) and Trimmomatic (version 0.40) were utilized to eliminate unreliable portions (erroneous bases) from given reads before aligning them with the human reference genome from the UCSC Genome Browser database (http://genome.ucsc.edu)[4,5]. Subsequently, STAR (version 2.7.10b) was used to align the trimmed scRNA-seq data to the reference human genome. The human genome annotation files from the UCSC Genome Browser database and annotations for 3220 HERVs were integrated for downstream quantitative analysis of HERVs[6].

3. Quality control and cell type determination

The cell count matrix for genes (including HERVs) were imported into the R package Seurat (version 4.3.0) for subsequent analysis[7]. We excluded cells with gene count <200 and >8000, removed genes expressed in less than 3 cells, and eliminated cells expressing mitochondrial genes by more than 5%. After applying these filtering criteria, the gene-cell count matrix was normalized using the NormalizeData () function. The 2000 most variable feature genes were determined, which exhibited substantial differences between cells, through the FindVariableFeatures () function for downstream analysis. PCA dimensionality reduction of the dataset was performed. The important principal components were identified by the JackStrawPlot () function through cell clustering and Uniform Manifold Approximation and Projection (UMAP)[7]. Cell types were defined by identifying marker genes through differential expression analysis[1]. Hematopoietic stem and progenitor cells (HSPCs) encompass hematopoietic stem cells and multipotent progenitors (HSCs/MPPs), lymphoid-primed multipotent progenitors (LMPPs), megakaryocyte and erythroid progenitors (MEPs), multipotent lymphoid progenitors (MLPs), and eosinophil, basophil, and mast cell progenitors (EBMs), as well as two neutrophil progenitor groups (Neu1 and Neu2), and two monocyte/dendritic progenitor subgroups (MD1 and MD2).

4. Differential expression analysis

Deseq2 (version 1.42.0) was employed to identify differentially expressed HERVs between AA and HC cells[8]. HERVs with fold-change greater than 1 and adjusted P<0.05 were determined to be significantly differentially expressed.

5. HERV expression analysis

The chi-square test was applied to adjust for cell sampling bias in each patient by determining the ratio of observed cell counts to the random expected (RO/E)[9]. This ratio was used to adjust for the expression levels of predicted HERVs versus actual levels of HERVs.

6. Weighted gene correlation network analysis

Weighted gene co-expression network analysis (hdWGCNA, version 0.2.2) of single-cell data was conducted, as developed by Morabito et al[10]. hdWGCNA is a novel algorithm that provides a highly modular approach to construct co-expression networks across multiple scales of cellular and spatial hierarchical structures, making it particularly suitable for scRNA-seq data. Based on the established Seurat object, we employed the KNN technology to aggregate the average or total expression of cell groups, generating a sparse meta-cellular gene expression matrix. We constructed a co-expression network with a soft threshold of 6 for subsequent analysis. All standard downstream analyses were performed according to the official workflow (<https://smorabit.github.io/hdWGCNA/index.html>).

7. Identification of genes and putative pathways associated with HERV families

To assess the potential effects of HERVs (*P*<0.05, log2FC >1) on cellular functions, genes within the same modules as HERVs were identified based on hdWGCNA. Gene Ontology (GO) enrichment analysis of these genes was performed to identify the enriched biological functions.

8. Cell communication

To analyze intercellular communication, we employed CellChat (version 1.5.0), a public repository for ligands, receptors, cofactors, and their interactions[11]. The versatile and user-friendly toolkit CellChat enables the discovery of novel intercellular communication and the construction of intercellular communication networks. For the analysis of cell interactions, expression levels were assessed relative to the total reads mapped to the same group of coding genes across the entire transcriptome. The expression levels within each single-cell cluster were averaged.

9. Single-cell trajectory construction

The HSPC trajectory was generated with the slingshot algorithm (version 2.4.0), inferring changes in gene and HERV expression in pseudo-time. The overall trajectory direction was controlled by the K-Means method. The associationTest () function was employed to determine HERVs that changed along the developmental trajectory[12].

**References：**

1. Zhu C, Lian Y, Wang C, Wu P, Li X, Gao Y, et al. Single-cell transcriptomics dissects hematopoietic cell destruction and T-cell engagement in aplastic anemia. Blood. 2021;138:23–33.

2. Tokuyama M, Kong Y, Song E, Jayewickreme T, Kang I, Iwasaki A. ERVmap analysis reveals genome-wide transcription of human endogenous retroviruses. Proceedings of the National Academy of Sciences. 2018;115:12565–72.

3. Andrews, S. (2010). FastQC: A Quality Control Tool for High Throughput Sequence Data [Online].

4. Martin M. Cutadapt removes adapter sequences from high-throughput sequencing reads. EMBnet.journal. 2011;17:10–2.

5. Bolger AM, Lohse M, Usadel B. Trimmomatic: a flexible trimmer for Illumina sequence data. Bioinformatics. 2014;30:2114–20.

6. Dobin A, Davis CA, Schlesinger F, Drenkow J, Zaleski C, Jha S, et al. STAR: ultrafast universal RNA-seq aligner. Bioinformatics. 2013;29:15–21.

7. Hao Y, Hao S, Andersen-Nissen E, Mauck WM, Zheng S, Butler A, et al. Integrated analysis of multimodal single-cell data. Cell. 2021;184:3573-3587.e29.

8. Love MI, Huber W, Anders S. Moderated estimation of fold change and dispersion for RNA-seq data with DESeq2. Genome Biology. 2014;15:550.

9. Wang J, Ren M, Yu J, Hu M, Wang X, Ma W, et al. Single-cell RNA sequencing highlights the functional role of human endogenous retroviruses in gallbladder cancer. eBioMedicine [Internet]. 2022 [cited 2023 Aug 29];85. Available from: https://www.thelancet.com/journals/ebiom/article/PIIS2352-3964(22)00501-1/fulltext

10. Morabito S, Miyoshi E, Michael N, Shahin S, Martini AC, Head E, et al. Single-nucleus chromatin accessibility and transcriptomic characterization of Alzheimer’s disease. Nat Genet. 2021;53:1143–55.

11. Jin S, Guerrero-Juarez CF, Zhang L, Chang I, Ramos R, Kuan C-H, et al. Inference and analysis of cell-cell communication using CellChat. Nat Commun. 2021;12:1088.

12. Street K, Risso D, Fletcher RB, Das D, Ngai J, Yosef N, et al. Slingshot: cell lineage and pseudotime inference for single-cell transcriptomics. BMC Genomics. 2018;19:477.

**Figure S1: Single-cell landscape and HERV expression in the AA and HC groups. A** Heatmap illustrating the expression of typical marker genes for major cell types. **B** Relative proportions of major cell types in AA cases and HCs. **C** Dot plot depicting the distribution of major cell types, as estimated by RO/E, across different tissue types. The ratio of observed to expected cell counts is shown for each cell type. **D** Intersection of upregulated and downregulated HERVs in HSC/MPP and MEP cell clusters.

**Figure S2: Volcano plots of differential HERVs in distinct subtypes of HSPCs. A-I:** Volcano plots depicting differential expression of HERVs in 9 subtypes of HSPCs. Criteria for selection were *P*<0.05 and log_2_ |fold change| >1.

**Figure S3: Co-expression analysis of HERVs in HSPCs. A:** Determination of the soft-thresholding parameter for co-expression analysis. **B:** Construction of a co-expression network based on the optimal soft-threshold, grouping genes and HERVs into distinct modules.

**Figure S4: T cell clustering. A:** UMAP projection of bone marrow CD8^+^ T cells from AA cases and HCs, color-coded by cell type. **B:** Heatmap depicting the expression of typical marker genes for major cell types in CD8^+^ T cells. **C:** Relative proportions of major cell types in CD8^+^ T cells from AA cases and HCs. **D:** UMAP projection of bone marrow CD4^+^ T cells from AA cases and HCs, color-coded by cell type. **E:** Heatmap illustrating the expression of typical marker genes for major cell types in CD4^+^ T cells. **F:** Relative proportions of major cell types in CD4^+^ T cells from AA cases and HCs.

**Figure S5: Volcano plots of differential HERVs in distinct subtypes of T cells. A-C:** Volcano plots illustrating differential expression of HERVs in 3 subtypes of CD8^+^ T cells. Criteria for selection were *P*<0.05 and log_2_ |fold change| >1. **D-F:** Volcano plots showing differential expression of HERVs in 3 subtypes of CD4^+^ T cells. Criteria for selection were *P*<0.05 and log_2_ |fold change| >1.

**Figure S6:** **Correlations between differential HERVs in HSC/MPP and MEP cell clusters and HLA expression in AA. A:** Density plot illustrating the expression densities of HLA-A, HLA-B, HLA-DRB1, and HLA-DRB5 loci in cell clusters from untreated AA patients and control samples. The red and blue boxes indicate the HSC/MPP and MEP cell clusters.

**Figure S7: Pseudotemporal analysis of HERVs and HLA in HSPCs from AA cases and HCs. A:** Pseudotemporal analysis trajectory of HSPCs, with cell subtypes referenced from Figure 1A. **B:** Expression patterns of differentially expressed HERVs in HSC/MPP and MEP from AA cases and HCs along trajectory 3.

**Supplemental Figure 1**


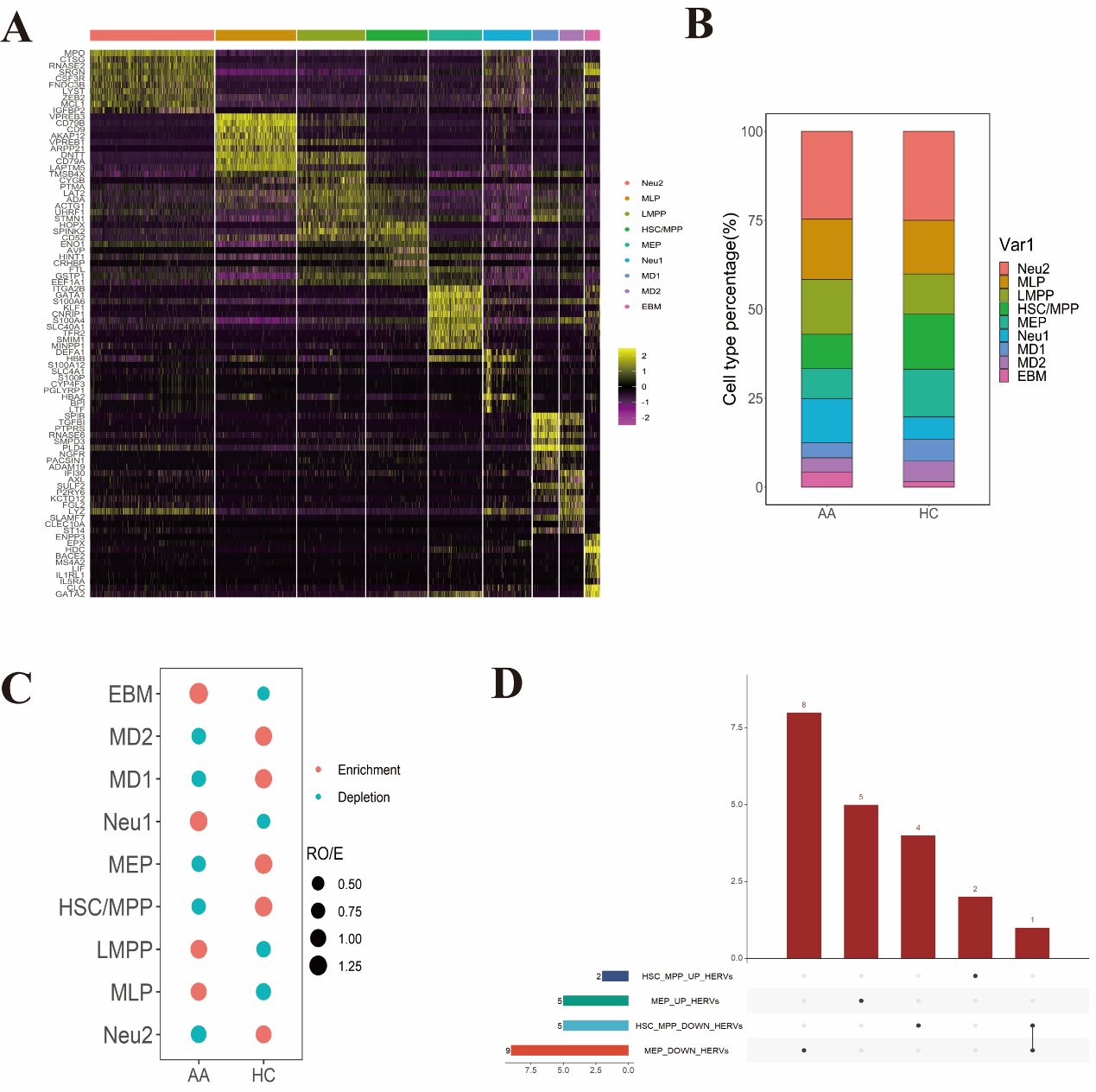


**Supplemental Figure 2**

**
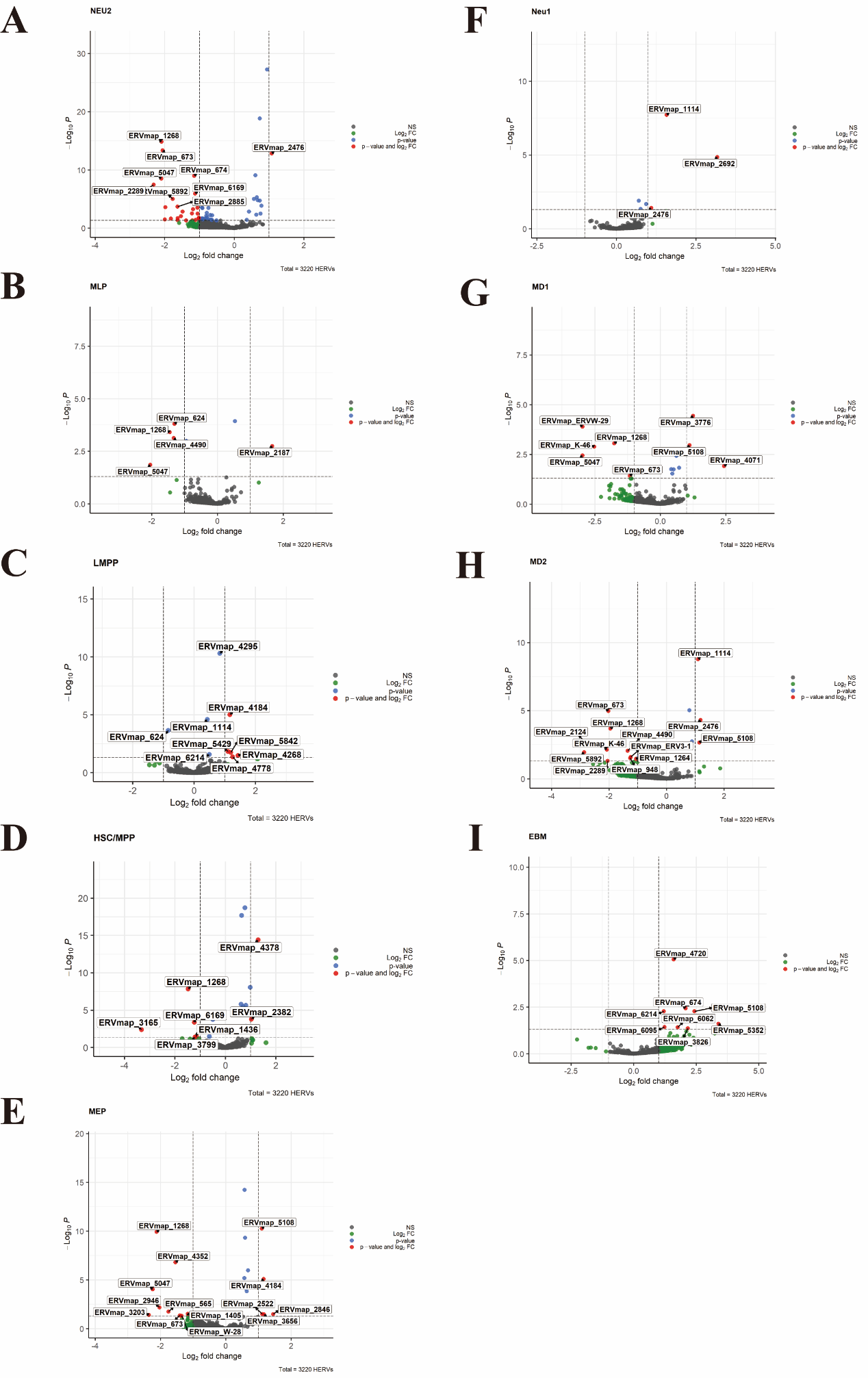
**

**Supplemental Figure 3**

**
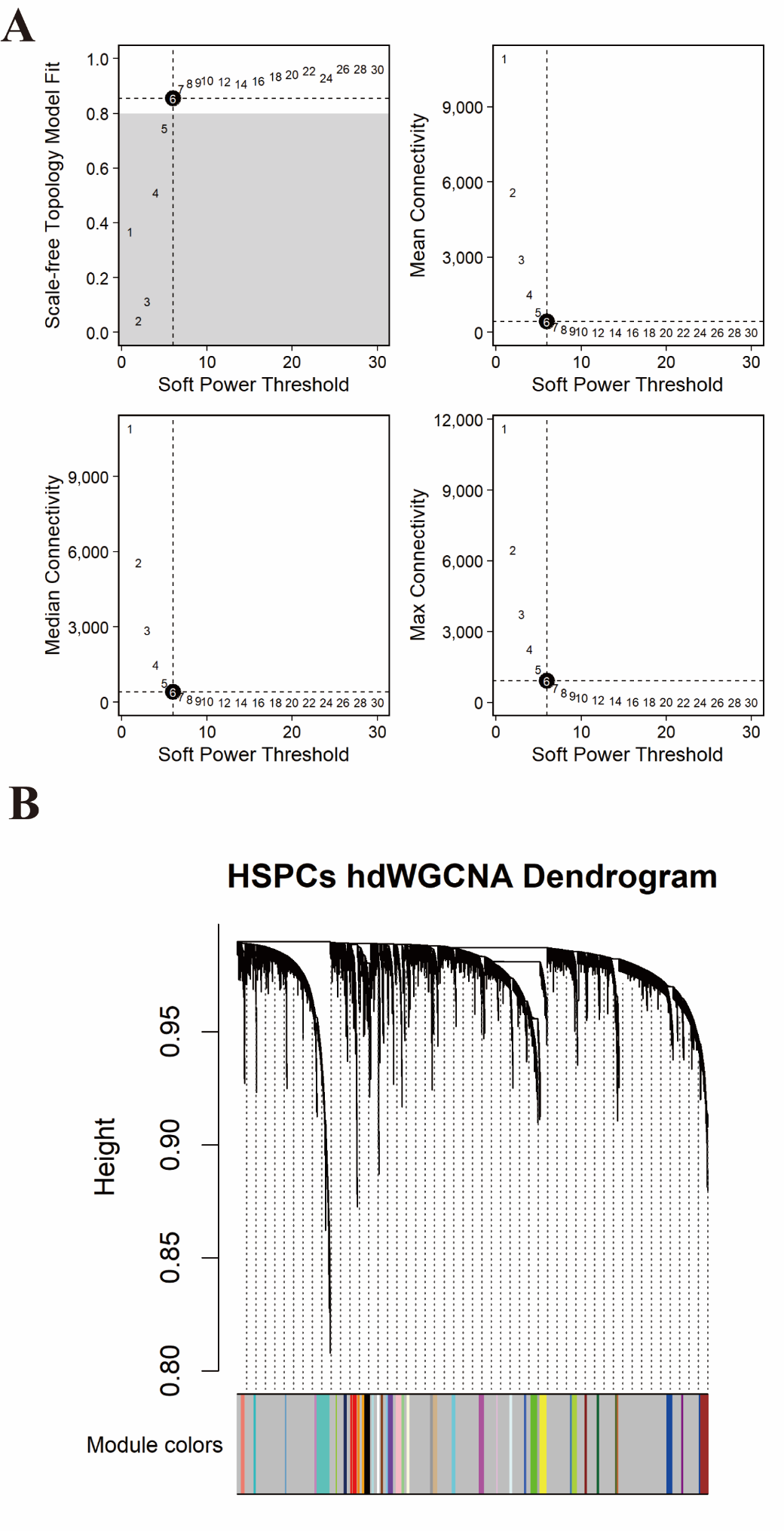
**

**Supplemental Figure 4**


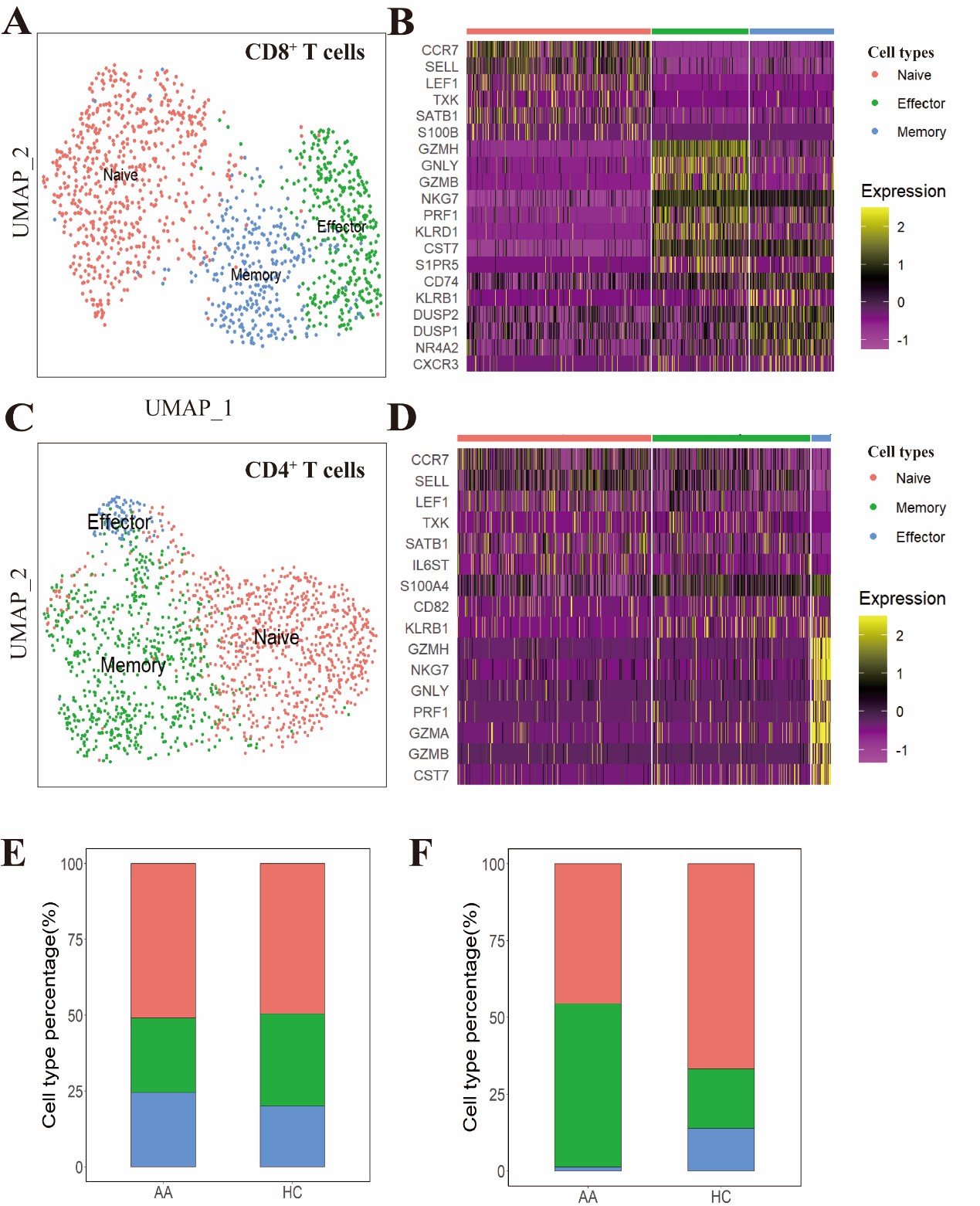


**Supplemental Figure 5**

**
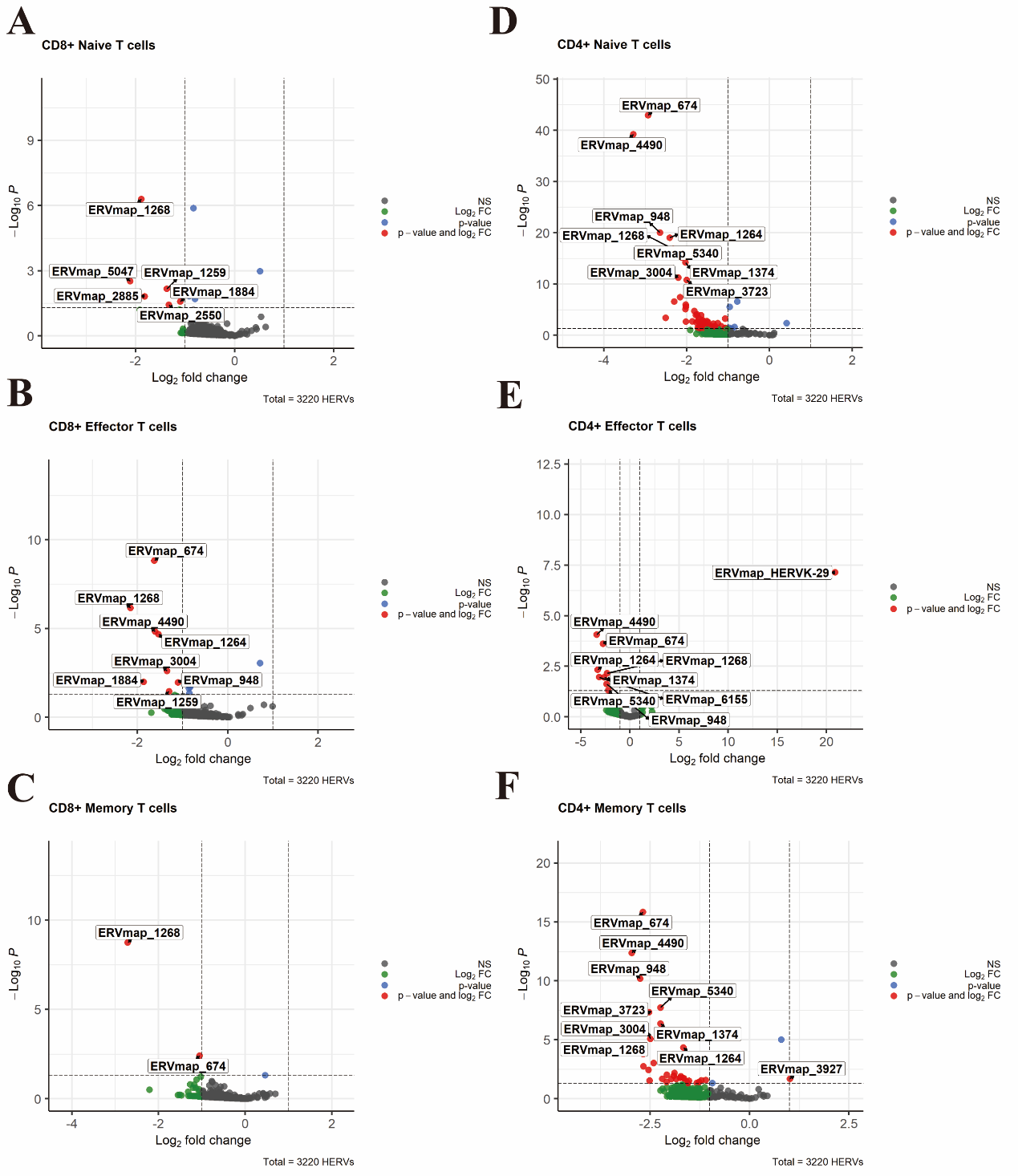
**

**Supplemental Figure 6**

**
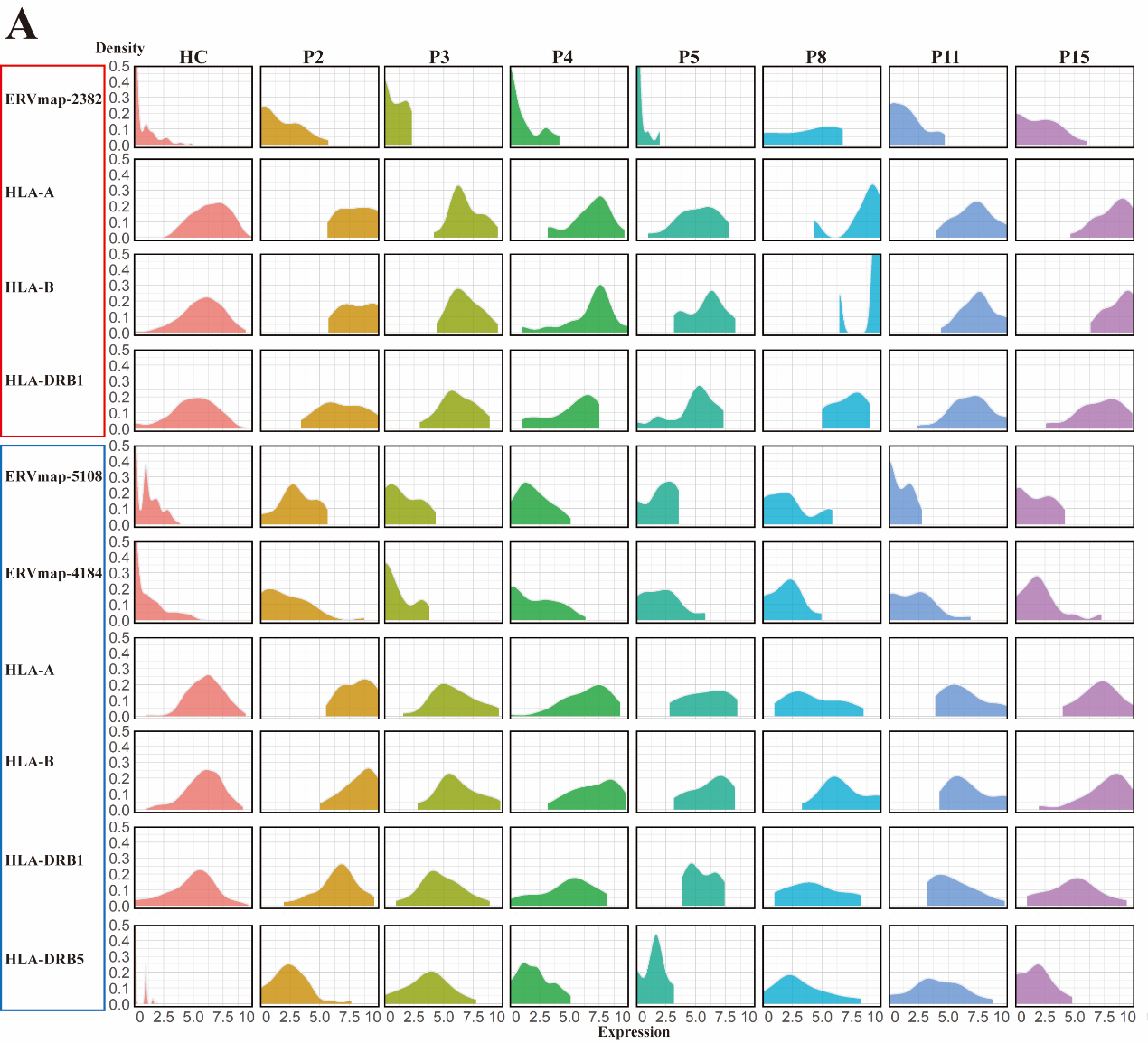
**

**Supplemental Figure 7**

**
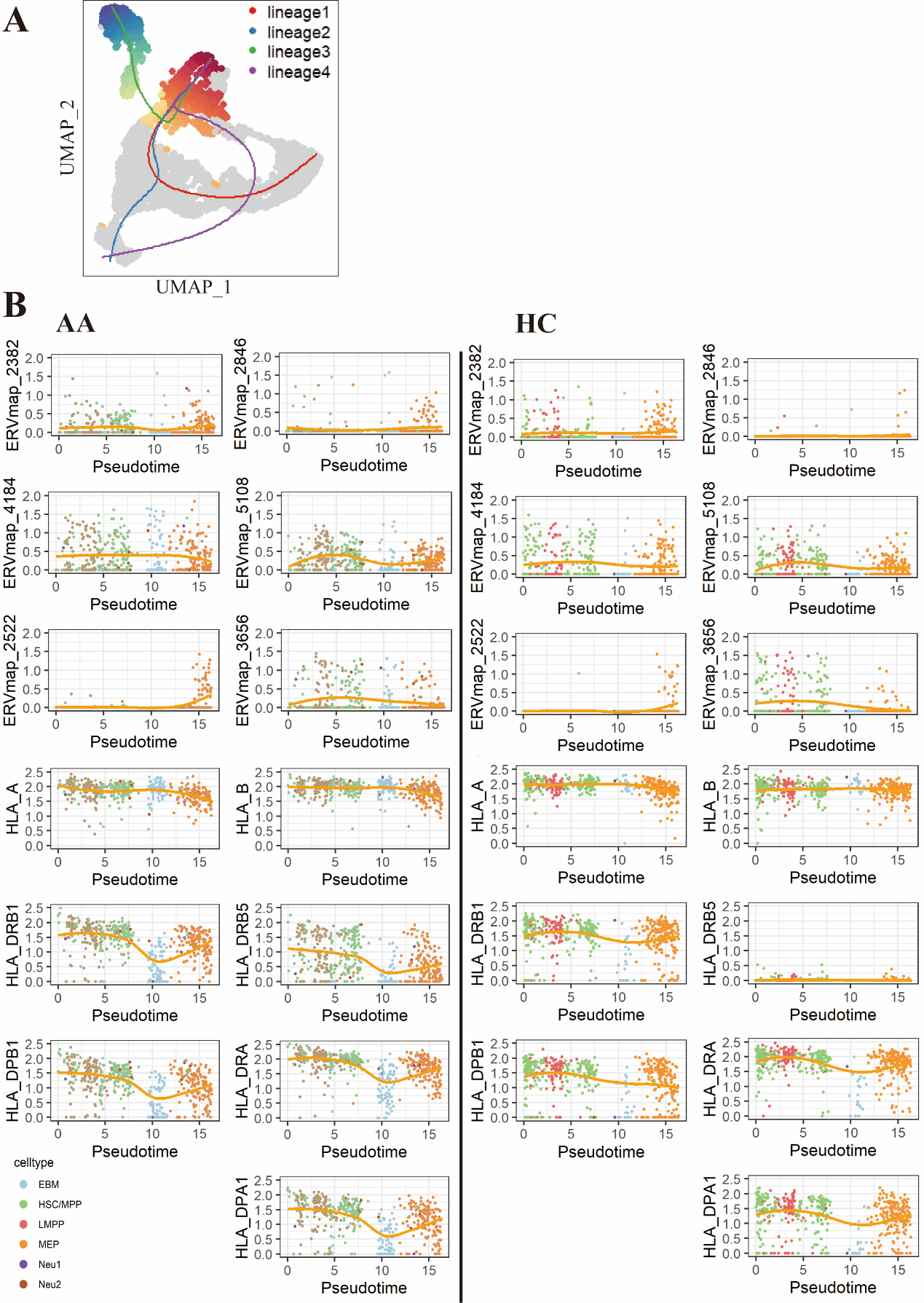
**
